# Editorial: Environmental stressors, multi-hazards and their impact on health

**DOI:** 10.3389/fpubh.2023.1231955

**Published:** 2023-07-11

**Authors:** Oyelola A. Adegboye, Faith O. Alele, Maru E. Castellanos, Anton Pak, Theophilus I. Emeto

**Affiliations:** ^1^Menzies School of Health Research, Charles Darwin University, Darwin, NT, Australia; ^2^Public Health and Tropical Medicine, College of Public Health, Medical and Veterinary Sciences, James Cook University, Townsville, QLD, Australia; ^3^World Health Organization Collaborating Center for Vector-Borne and Neglected Tropical Diseases, College of Public Health, Medical and Veterinary Sciences, James Cook University, Townsville, QLD, Australia; ^4^Australian Institute of Tropical Health and Medicine, James Cook University, Townsville, QLD, Australia; ^5^Centre for the Business and Economics of Health, The University of Queensland, Brisbane, QLD, Australia

**Keywords:** environmental stressor, air pollution, noise—exposure, temperature, heatwaves, human health, exposure sciences, environmental health

Environmental stressors, such as air pollution, noise pollution, and chemical exposure, can adversely affect human health by increasing the risk of chronic diseases and mortality (1–5). The sixth assessment report of the United Nations' Intergovernmental Panel on Climate Change reported that the global temperature is projected to reach or exceed 1.5°C of warming over the next 20 years (6), exacerbating exposure to environmental stressors. Air pollution alone is estimated to cause 4.2 million deaths annually (7), and most of the world's population (99%) is exposed to air quality levels that exceed the WHO Air Quality Guidelines (8).

Moreover, environmental stressors often co-occur, creating multi-hazard scenarios that can have synergistic or cumulative impacts on human health. For example, exposure to air and noise pollution can increase respiratory and cardiovascular disease risk (9–12). This Research Topic aims to provide an overview of the current state of knowledge on the relationship between environmental stressors and health, focusing on multi-hazard situations. The Research Topic includes seven original articles investigating and quantifying the health effects of various environmental stressors and multi-hazards using different methods and data sources.

Ahn and Min used data from the National Health Insurance Service in South Korea to investigate how meteorological factors and air pollutants affected epistaxis occurrence in 46,628 patients from different age groups. They found that epistaxis incidence was highest among people < 18 years (41.99%) and lowest among people aged >70 years (5.74%), and that it varied by month and age group, peaking in January and dropping in July. Only PM_10_ and SO_2_ were associated with epistaxis among the air pollutants, especially in people < 18 and 18–40 years. The association between air pollutants and epistaxis was influenced by seasonal variations and differed from previous studies, suggesting the need to consider specific pollutant concentrations, climate variables, and age in assessing their effects on epistaxis occurrence. The association also differed by age group, with wind speed being more important for the pediatric group and temperature is more important for the adult groups, indicating age-specific associations between these factors and epistaxis occurrence.

Guo et al. examined how meteorological and environmental changes affected bronchial asthma in children < 14 years in a Shanghai hospital. They screened 9,322 children with acute respiratory tract infections and found a significant difference in bronchial asthma hospitalization before (11.2%, *n* = 668) and after (9.6%, *n* = 319) the COVID-19 pandemic. They attributed the decline in acute asthma exacerbations after the pandemic to lifestyle changes and preventive measures such as home learning, reduced public gatherings, frequent handwashing, regular disinfection, and face masking. These measures likely helped prevent or reduce respiratory infections. The authors found no significant change in meteorological factors before and after the pandemic but observed notable changes in environmental factors. After the pandemic, pollutants such as NO_2_, SO_2_, PM_2.5_, and PM_10_ decreased, while O_3_ levels increased. The study showed a positive correlation between PM_2.5_ and acute asthma exacerbations after the pandemic. PM_2.5_ exposure is associated with an increased risk of asthma hospitalization. Conversely, temperature and O_3_ had significant negative effects on the number of acute asthma exacerbations.

In another study, Huang et al. examined the relationship between hostility level (a proxy for negative emotions) and urban summer high temperatures and outdoor activity duration in 931 middle-aged and older people (40+ years) in Beijing using the Chinese Positive and Negative Affect Schedule questionnaire (13). The questionnaire data revealed a balanced gender ratio among respondents, with similar hostility levels between males and females, while people in the 40–49 age group had the highest mean hostility score and those engaged in outdoor activities had the lowest hostility score. The study assessed the emotional health risk (hostility) based on temperature standards and found a rapid decline in low-risk areas, with only a few remaining in specific green space locations, while medium-risk areas expanded significantly, covering 97.58% of the study area. High-risk areas affected by urban heat changed slower but gradually expanded, posing a greater threat to residents' emotional health, especially in highly urbanized regions. The study showed a positive correlation between hostility and average daytime temperature, indicating that higher temperatures increase the risk of heat waves, negatively affecting residents' emotional health. Extreme heat caused stress, fatigue, and negative emotions such as depression, anger, pain, and hostility, while extremely low temperatures reduced negative emotions. A fitted non-linear polynomial second-order model revealed a “*U*-shape” relationship, indicating that hostility decreased gradually with shorter durations but increased with longer durations. The urban environment, with its complexity and low comfort, elicited negative emotions such as tension and hostility. However, as outdoor activity duration increased, the human body gradually adapted to the high-temperature environment, reducing hostility.

Using a time series study, Song et al. evaluated the relationship between ambient carbon monoxide (CO) exposure and the risk of hospitalization for common respiratory diseases. They analyzed daily hospitalization data from January 2016 to December 2020 from the largest hospital in Ganzhou with potential confounding factors such as age, gender, and season to identify susceptible populations. They recorded 72,430 cases of respiratory diseases during the study period. Using the generalized additive model, the reported significant positive exposure–response relationships between ambient CO exposure and the risk of hospitalization for respiratory diseases. For every 1 mg/m^3^ increase in CO concentration (lag0–2), the risk of hospitalization increased for total respiratory diseases, asthma, chronic obstructive pulmonary disease, lower respiratory tract infection and influenza-pneumonia. The study showed a clear detrimental effect of CO on respiratory health and a linear exposure–response relationship, suggesting that even small increases in CO levels can lead to a higher risk of hospitalization. The study also highlights the influence of season and gender as effect modifiers in the relationship between CO exposure and respiratory disease hospitalisations. The differential susceptibility of women to CO exposure-associated hospitalisations for asthma and lower respiratory tract infections underscores the importance of considering gender-specific vulnerability in respiratory health outcomes.

Sisto et al. explored the potential of microRNAs (miRNAs) as biomarkers of early occupational hearing impairment caused by combined exposure to noise and volatile organic compounds (VOCs) in painters. Occupational hearing impairment is a common and serious health issue that affects millions of workers worldwide. Noise and ototoxic chemicals such as VOCs can damage the inner ear, leading to permanent hearing loss. However, the damage mechanisms and biomarkers for early detection and prevention remain unclear. miRNAs are small non-coding RNAs that modulate gene expression and involve various biological processes, such as development, differentiation, apoptosis, and stress response (14). The authors applied an unsupervised artificial neural network to visualize the hidden relationships between the variables and to identify clusters of subjects with similar features. They found that miRNA expression profiles can differentiate between exposed workers with different levels of hearing impairment and between exposed workers and controls. They also showed that exposure to both noise and VOCs results in a significant worsening of hearing loss compared to noise exposure alone. Their data indicated that miRNAs significantly correlate with hearing loss (HL) and distortion product otoacoustic emissions (DPOAE) levels, suggesting that miRNAs are specific biomarkers of susceptibility to neurotoxic exposure beyond age and noise exposure alone. They identified 12 differentially expressed miRNAs correlated with audiological variables and discriminated between two groups based on DPOAE level and HL, suggesting a potential diagnostic tool for hearing impairment due to VOCs exposure.

Qu et al. reported the results of a cross-sectional study conducted in two cities in southern China from July to August 2018. They investigated the association between exposure to cadmium and/or lead and diabetes mellitus type 2 (T2DM) and obesity in 1,274 residents from the Dapeng community in Shenzhen City and Hengq community in Zhuhai City. They estimated exposure to heavy metals by measuring the levels of cadmium and lead in drinking water and soil samples and then calculating the average daily dose (ADD) of these metals. The outcome variables were T2DM as self-reported by the participants and obesity measures-waist circumference (WC), waist-to-hip ratio (WHR), and body mass index (BMI) as measured by the researchers. They built several adjusted regression models to evaluate the association between ADD of lead/cadmium and health outcomes. The main findings showed no association between ADD of lead/cadmium and T2DM in this population. For obesity measures, the results were mixed. Exposure to lead was associated with lower WC, WHR, and a lower likelihood of being overweight. However, exposure to cadmium was not associated with WHR, and there was no conclusive result for WC and BMI.

A study by Li et al. examined the relationships between plasma concentrations of 2,992 proteins and working in very cold, hot, dusty, and noisy workplaces, as well as exposure in the workplace to chemicals and other fumes. They also assessed the relationships between plasma proteins potentially affected by workplace environments and the risks of disease to estimate whether the variation of these plasma protein levels potentially affects disease risks. They utilized a Mendelian randomization design to identify proteins that could be used as biomarkers of health status. Working in a noisy environment was associated with decreased concentrations of insulin-like growth factor-binding protein 1, secretogranin-3, type 2 lactosamine alpha-2,3-sialyltransferase, and unc-5 netrin receptor D levels. In contrast, plasma concentrations of CMP-N-acetylneuraminate-beta-galactosamide-alpha-2,3-sialyltransferase 2 (ST3GAL2) were increased in association with a noisy work environment. Exposure to chemical fumes up-regulated 13 plasm proteins. Down-regulated proteins were growth-regulated alpha protein and ephrin-A3, and trefoil factor 1 (TFF1). Transmembrane protein 132A (TMEM132A) and carbonic anhydrase-related protein 10 (CA10) were increased following exposure to secondhand cigarette smoke, while neurensin-1 and pituitary adenylate cyclase-activating polypeptide 38 were decreased. Exposure to diesel exhaust altered the plasma concentrations of leukocyte immunoglobulin-like receptor subfamily B member 2 and teneurin-4 (TENM4). These proteins were associated with the risk of at least one disease. The evidence suggests that harsh work-related environments may alter the concentration of plasma proteins, and these proteins may be biomarkers to monitor occupational hazard risk.

In conclusion, this Research Topic highlights the impact of various environmental stressors on different health issues. To protect human health from these stressors, a comprehensive approach that addresses the multiple hazards people are exposed to and implements policies and regulations limiting exposure is important. To reduce the impact of environmental stressors, our environment should be considered not only as a source of resources but also as a determinant of human health. The evidence shows that more research is needed to identify biomarkers to monitor environmental risks. Strategies to reduce environmental stressors and protect our health and the health of future generations are required. These include increasing public awareness and health education, promoting clean energy sources, prioritizing vulnerable groups, and implementing urban planning measures.

## Author contributions

OA: conceptualization, investigation, and writing—original draft preparation, review, and editing. FA and TE: investigation and writing—original draft preparation, review, and editing. MC and AP: writing—original draft preparation, review, and editing. All authors contributed to the article and approved the submitted version.
